# Editorial: Neonatal infections and the developing neonatal immune system: current evidence and research gaps to fill

**DOI:** 10.3389/fped.2023.1243752

**Published:** 2023-07-17

**Authors:** Carlo Pietrasanta, Maria Giulia Conti

**Affiliations:** ^1^ NICU, Department of Woman, Child and Newborn, Fondazione IRCCS Ca' Granda Ospedale Maggiore Policlinico, Milan, Italy; ^2^ Department of Clinical Sciences and Community Health, University of Milan, Milan, Italy; ^3^ Department of Maternal and Child Health, Policlinico Umberto I, Sapienza University of Rome, Rome, Italy

**Keywords:** neonatal immune response, immune ontogeny, neonatal infections, immunology & infectious diseases, neonatal immunology

**Editorial on the Research Topic** Neonatal infections and the developing neonatal immune system: current evidence and research gaps to fill

Infections during the first weeks of life still represent a major cause of morbidity, both short and long term, and mortality worldwide. According to the World Health Organization, neonatal infections result in over 550,000 neonatal deaths every year, but despite this significant burden of disease, therapeutic options for neonatal infections, are still limited. For example, apart from antibiotics and supportive care, we currently lack other effective therapeutic tools to prevent and treat neonatal sepsis Pietrasanta et al. This is partially due to our incomplete understanding of the neonatal immune function and response, especially during infections (1, 2). Complex and unique mechanisms regulate the interaction between the host and environmental antigens in early life, starting during pregnancy: indeed, the neonatal immune system must maintain a continuous balance between tolerance towards maternal antigens *in utero* and commensal microorganisms after birth, and the ability to mount effective humoral and cell-mediated responses against invading pathogens (3). This balance is especially critical at the level of the respiratory and gastrointestinal mucosae, that are the major sites of contact between microorganisms and the immune system. Thus, up-to-date clinical and mechanistic data linking the occurrence of neonatal infections and the immunological mechanisms of host-microorganisms interaction are needed to improve our understanding of neonatal infections. This collection of scientific articles deals with the issue, and encompasses 4 original articles, 1 brief report, 2 mini reviews and 1 systematic review.

Two group of researchers focused their attention on neonatal sepsis. In their systematic review, **Zhang et al**. summarized the utility of peripheral blood leucocyte ratios as biomarkers in neonatal sepsis, highlighting the extreme heterogeneity of published studies and the need to combine multiple hematological parameters to obtain the best diagnostic performance for neonatal sepsis. Overall, at present, the immature-to-mature neutrophil ratio (IMR) seems the single most accurate biomarker for the diagnosis of neonatal sepsis, but combinations of biomarkers constantly perform better than single ones.

**Ganji et al**. from Canada analyzed instead the importance of family care to reduce the incidence of neonatal sepsis, possibly through increased breast milk intake and more physiological neurodevelopment of neonates admitted to neonatal intensive care units (NICUs). The 5 studies analyzed reported a reduction of 25% in neonatal sepsis rates, a result that highlights the importance of families to significantly improve the health and outcomes of critical neonates.

Apart from bacterial sepsis, viral infections are key contributors to neonatal morbidity and mortality, in part because treatment weapons against these pathogens are still limited and their pathogenesis is far from being clarified. This has been elegantly summarized by **Sakleshpur and Steed** in their review article on the immune response against influenza virus in early life. **Dauby and Flamand** contributed this reasoning adding another important factor that might affect neonatal immune response after birth, which is the occurrence of maternal respiratory infections (such as COVID-19, or influenza itself) during pregnancy. The last contribution on this issue came from **De Rose et al.** who compared the performance of three different clinical scores for the prediction of bronchiolitis severity in neonates and infants admitted to neonatal units.

Viral diseases in early life are not exclusively respiratory: indeed, systemic infections such as those caused by Cytomegalovirus (CMV) or enteroviruses are not rare in neonates, with wide a range of severity. **Chen et al.** reinforced the evidence that CMV infection acquired after birth is rarely harmful for term born and late-preterm neonates: in this topic, the authors report no impact of postnatal CMV on body growth or liver function at 1 year of age, and reassured on the opportunity to promote breastfeeding in these populations of neonates even in case of maternal CMV infections. Conversely, acquired infection by the enterovirus Echovirus type 11 can be associated with devastating neonatal outcomes such as haemorrhage-hepatitis syndrome: **Wang et al.** from China reported data from a retrospective multicenter cohort of infected neonates in China, clarifying possible predisposing factors and early predictors of infection severity.

Finally, **Athikarisamy et al.** from Western Australia investigated the association between prenatal inflammation of the placenta, frequently due to intrauterine infections, and severe retinopathy of prematurity (ROP), an ocular complication of preterm birth for the occurrence of which inflammation may play a significant role. Interestingly, after adjusting for several confounders, the presence of both mild and severe placental inflammation was associated with reduced odds of severe ROP, while gestational age, supplemental oxygen and the need for postnatal steroids showed the strongest association with ROP.

## Conclusion

This special issue provides useful insights on the progresses made in the field of neonatal immune and inflammatory response to microbial infections. Each of the summarized contributions broadens our understanding of several aspects of this condition. Nonetheless, several functional and anatomical characteristics of the neonatal immune system, as well as the mechanisms driving a physiological interaction with microbial components in early life, have yet to be clarified through clinical and experimental research to be conducted in the future.
